# The association between cingulate cortex glutamate concentration and delay discounting is mediated by resting state functional connectivity

**DOI:** 10.1002/brb3.74

**Published:** 2012-07-16

**Authors:** Lianne Schmaal, Anna E Goudriaan, Johan Meer, Wim Brink, Dick J Veltman

**Affiliations:** 1 Amsterdam Institute for Addiction Research, Department of Psychiatry, Academic Medical Center, University of AmsterdamAmsterdam, The Netherlands; 2 Arkin Mental Health InstituteAmsterdam, The Netherlands; 3 Department of Sleep and Cognition, Netherlands Institute for Neuroscience, Royal Netherlands Academy of Arts and SciencesAmsterdam, The Netherlands; 4 Department of Psychiatry, VU university medical centerAmsterdam, The Netherlands

**Keywords:** Anterior cingulate cortex, delay discounting, glutamate, impulsive decision making, magnetic resonance spectroscopy, resting state fMRI

## Abstract

Humans vary in their ability to delay gratification and impulsive decision making is a common feature in various psychiatric disorders. The level of delay discounting is a relatively stable psychological trait, and therefore neural processes implicated in delay discounting are likely to be based on the overall functional organization of the brain (under task-free conditions) in which state-dependent shifts from baseline levels occur. The current study investigated whether delay discounting can be predicted by intrinsic properties of brain functioning. Fourteen healthy male subjects performed a delay discounting task. In addition, resting state functional magnetic resonance imaging (fMRI) and magnetic resonance spectroscopy (¹H MRS) were used to investigate the relationship between individual differences in delay discounting and molecular and regional measures of resting state (baseline) activity of dorsal anterior cingulate cortex (dACC). Results showed that delay discounting was associated with both dACC glutamate concentrations and resting state functional connectivity of the dACC with a midbrain region including ventral tegmental area and substantia nigra. In addition, a neural pathway was established, showing that the effect of glutamate concentrations in the dACC on delay discounting is mediated by functional connectivity of the dACC with the midbrain. The current findings are important to acknowledge because spontaneous intrinsic brain processes have been proposed to be a potential promising biomarker of disease and impulsive decision making is associated with several psychiatric disorders.

## Introduction

Humans vary considerably in their ability to delay gratification and maladaptive levels of impulsive decision making are a common feature in various psychiatric disorders, including substance use disorder, attention deficit hyperactivity disorder (ADHD), conduct disorder, bipolar disorder, and pathological gambling (Moeller et al. 2001). Impulsive decision making is reflected by an increased preference for (smaller) immediate rewards over (larger) delayed rewards and often assessed by delay discounting paradigms. Impulsive decision making is a relatively stable psychological trait that is at least partly attributable to genetic differences, although state-dependent shifts from baseline (trait) levels can occur (for a review see Peters and Buchel 2011). Given the trait-like characteristics of impulsive decision making, it can be argued that individual differences in delay discounting can be predicted by intrinsic properties of brain functioning, such as brain metabolites and spontaneous fluctuations in blood oxygen level-dependent (BOLD) activity.

In recent years, considerable progress has been made in unraveling the underlying neurobiology of impulsivity. On a molecular level, various neurotransmitter systems have been implicated in impulsive decision making (Winstanley et al. 2006; Pattij and Vanderschuren 2008). Historically, the focus has been on the role of dopaminergic and serotonergic neurotransmission underlying impulsivity, but more recently, evidence for a role of glutamate has been found. In animals, a metabotropic glutamate 1 receptor antagonist significantly increased preference for large reward at longer delay values in the delay discounting task (DDT; Sukhotina et al. 2008). In humans, an association has been found between glutamate concentrations in the dorsal anterior cingulate cortex (dACC) and self-reported impulsivity (Hoerst et al. 2010). On the level of regional brain activity, resting state functional connectivity could provide an intermediate step between brain metabolite concentrations and behavior because it does not only probe specific cognitive functions as in task-related functional magnetic resonance imaging (fMRI), but may identify major functional networks that contribute to variability in behavior (Laird et al. 2011). For example, resting state functional connectivity reflected by brain regions showing similar patterns of spontaneous activation over time networks have been shown to predict the task-response properties of brain regions (De et al. 2005; Vincent et al. 2006) and predict individual performance variability in several cognitive domains (Hampson et al. 2006; Seeley et al. 2007; Zhu et al. 2011; Baldassarre et al. 2012). This indicates that individual differences in behavior are reflected in the brain's intrinsic functional architecture. Hence, resting state functional connectivity may offer a valuable tool for analyzing the functional basis of interindividual variation in impulsive decision making.

Neural processes implicated in trait impulsivity are likely to be based on the overall functional organization of the brain (under task-free conditions), in which state-dependent shifts from baseline levels occur to adapt decision making to a changing environment or changing cognitive demands. Therefore, the aim of this study was to further delineate the underlying neurobiology of impulsive decision making in healthy volunteers by combining MRI methods assessing resting state (baseline) brain processes at different levels. On a molecular level, localized proton magnetic resonance spectroscopy (¹H MRS) in the anterior cingulate cortex (ACC) was used to measure glutamate concentrations. In addition, resting state functional connectivity of the ACC was assessed as a regional measure of resting state activity. Moreover, a mediation analysis (Fig. S1) was conducted to establish a functional pathway from molecular properties of the dACC to impulsive decision making through resting state functional connectivity of the dACC with other brain regions. The dACC was chosen as our region of interest (ROI), because BOLD responses in the dACC play an important role in delay discounting (Hoffman et al. 2008; Marco-Pallares et al. 2010) and glutamatergic abnormalities in the dACC measured by ¹H MRS have been related to self-reported impulsivity (Hoerst et al. 2010).

## Methods

### Subjects

Fourteen nonsmoking healthy male subjects (mean age: 35, SD: 9.5 years) were recruited based on the following exclusion criteria: presence of *DSM-IV* diagnosis of psychiatric disorders; lifetime history of head injury with loss of consciousness for more than 5 min; neurological disorders; positive urine tests for alcohol, methadone, benzodiazepines, cocaine, amphetamines, marijuana, or opiates; unstable medical condition; estimated IQ below 80; any use of medication affecting the central nervous system; and MRI ineligibility due to nonremovable metal objects or claustrophobia. All subjects gave written informed consent to participate in this study, which was approved by the Medical Ethical Committee of the Academic Medical Center, University of Amsterdam.

### Procedure

Assessments took place in the afternoons. After informed consent was obtained, subjects' IQ was estimated using the Dutch version of the National Adult Reading Test (Schmand et al. 1991), followed by administration of the DDT which took approximately 10 min. After a short break of 15 min, subjects underwent a scanning session including T1-weighted images, gradient-echo echo-planar (EPI) images during rest and ¹H MRS (in that order).

### Delay discounting paradigm

A DDT (Wittmann et al. 2007) was included to assess impulsive decision making reflected by an increased preference for (smaller) immediate rewards over (larger) delayed rewards. In short, the subjects were asked to make a decision between a hypothetical immediate reward and a reward to be received in the future. The task consisted of six blocks of eight preference judgment trials. Within each block, the future reward was fixed, with a block specific delay in days, *d*, and reward magnitude in euro's, *x*, that is, (*d*, *x*) = (5, 506), (30, 476), (180, 524), (365, 512), (1095, 520), and (3650, 488) for blocks 1–6, respectively. The blocks were presented in random order. The immediate reward varied in magnitude from trial to trial within each block according to a rule to successively narrow the range of the magnitude of the immediate reward that was equally preferred to the delayed reward, resulting in an indifference point for every block. For a detailed description of the algorithm that was used to obtain the indifference points, the reader is referred to Wittmann et al. (2007). By plotting the indifference points against each of the six delays, an estimation of the steepness of delay discounting could be obtained for each subject. A hyperbolic discounting function is often utilized to describe the relationship between the subjective value of a reward as a function of the delay, however, because of a limited goodness-of-fit of the data to and a non-normal distribution of the parameters obtained by the hyperbolic discounting function, we assessed discounting behavior using the area under the curve (AUC) method (Myerson et al. 2001). We plotted the indifference points against the delay for all six blocks and calculated the AUC. Smaller AUC values represent steeper discounting rates, and thus higher impulsive decision making.

### Magnetic resonance spectroscopy (¹H MRS) acquisition and processing

**Figure 1:**
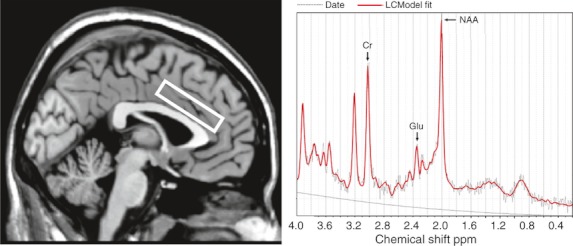
Voxel placement. Voxel placement in left dACC for localized single-voxel ¹H MRS and a representative spectrum of one subject. Cr, creatine; Glu, glutamate; NAA, *N*-acetylaspartate.

MRI and MRS data were obtained using a 3.0 T Intera MRI scanner (Philips Healthcare, Best, The Netherlands) equipped with a SENSE eight-channel receiver head coil. Three-dimensional T1-weighted images were collected in the sagittal plane using a gradient-echo sequence (repitition time (TR) = 9 ms; echo time (TE) = 3.5 ms; 170 slices; voxel size 1 × 1 × 1 mm; matrix size 256 × 256). Using these images, a single ¹H MRS voxel was placed in the left supracallosal ACC (Fig. 1). MRS was performed using a point resolved spectroscopy sequence (PRESS; TR = 2000 ms; voxel size 50 × 16 × 10 mm; 64 acquisitions) using a TE of 38 ms. A TE of 38 ms was chosen because reliable estimates of the glutamate signal with this echo time were obtained previously in our laboratory and it approximates the echo time reported in a study that found improved detection of glutamate with a TE of 40 ms (Mullins et al. 2008). Spectra were acquired using first order iterative shimming and water suppression was automatically performed by the scanner.

Spectra derived from ¹H MRS from 4.0 to 0.2 ppm were analyzed using LCModel (Linear Combination of Model spectra; Provencher 1993). LCModel is a user-independent analysis method that estimates metabolite concentrations by fitting the in vivo spectra to a set of previously acquired in vitro spectra (the basis set). Results are presented in institutional units approximating millimolar (ppm) concentration. We used the Cramér-Rao lower bounds (CRLB), a measure of the reliability of the fit, less than 20% for each individual peak as the quality criterion (Provencher 1993). The CRLBs for glutamate in all subjects were between 7% and 12%. Additional indicators for quality of the spectra were signal to noise ratio (mean = 16.64, SD = 2.53) and the full width half maximum (FWHM; mean = 0.05, SD = 0.02). Spectra of all subjects passed the quality control. Glutamate concentrations are given as their ratio to creatine (Glu/Cr). The ratio of glutamate concentration to creatine (Glu/Cr) was calculated with LCModel.

### Resting state functional MRI (rs-fMRI) acquisition and processing

For the resting state functional imaging data, subjects were instructed to keep their eyes closed, remain still, and to not fall asleep. A gradient-echo echo-planar (EPI) sequence sensitive to BOLD contrast (TR/TE = 2300 ms/25 ms, matrix size 64 × 64, voxel size 2.29 × 2.29 × 3 mm, 38 slices of 3 mm) was used to acquire 200 images. Anatomical imaging included a sagittal 3D gradient-echo T1-weighted sequence (TR/TE = 9 ms/3.5 ms, matrix size 256 × 256, voxel size: 1 × 1 × 1 mm; 170 slices).

Resting state fMRI (rs-fMRI) data were preprocessed using the Statistical Parametric Mapping package (SPM8; Welcome Department for Imaging Neuroscience, London, UK: http://www.fil.ion.ucl.ac.uk/spm). For each subject, EPI images were reoriented, realigned, and unwarped, correcting for movement and normalized into standardized Montreal Neurological Institute (MNI) space. To make sure that the results were not induced by an inadequate warping of the brainstem, we used the unified segmentation algorithm available in SPM to perform normalization during preprocessing. This has been shown to achieve good intersubject coregistration for brain areas, such as the striatum and the brainstem (Klein et al. 2009). Next, linear detrending was carried out using a software package named Resting-State fMRI Data Analysis Toolkit (REST, by SONG Xiao-Wei et al., http://resting-fmri.sourceforge.net).

Regional spontaneous activity was assessed in the left dorsal ACC by calculating the fractional amplitude of low frequency fluctuation (fALFF: Zou et al. 2008), as implemented in the REST toolkit. The ACC mask was defined by merging the individually placed spectroscopy voxel position in normalized space to correspond to the size and placement of the MRS voxel that was used for obtaining MRS spectra in the left dACC. The fALFF was calculated by transforming the time series for each voxel to a frequency domain (without bandpass filtering) using fast Fourier transform to obtain the power spectrum. As the power of a given frequency is proportional to the square of its amplitude in the original time series, the square root was calculated at each frequency of the power spectrum. The sum of the amplitude across 0.01–0.08 Hz was divided by that across the entire frequency range (0–0.25 Hz). The fALFF of each voxel was divided by the global mean fALFF value to standardize data across subjects and then a spatial smoothing transformation was conducted with an 8 mm Gaussian kernel.

For functional connectivity analyses, the preprocessed fMRI data were temporal bandpass filtered (0.01–0.08 Hz) and spatially smoothed with an 8 mm Gaussian kernel. To remove physiological nuisance, the global volume intensity, the global white matter, and cerebral spinal fluid (CSF) temporal signals were regressed from the time series (Fox et al. 2005). In addition, motion parameters were treated as nuisance and removed via regression. For functional connectivity (rs-FC) of the dACC with other brain regions, we defined a priori ROIs based on a literature review of neuroimaging studies using delay discounting paradigms. These ROIs encompassed the lateral prefrontal cortex (L_PFC), which is part of a cognitive control network involved in the DDT together with the ACC, the ventromedial PFC (vmPFC), the posterior cingulate cortex (PCC), the ventral striatum, and the midbrain including substantia nigra (SN) and ventral tegmental area (VTA) which have been implicated in the valuation of rewards, and the hippocampus and amygdala which have been implicated in prospection/future thinking during the DDT (Peters and Buchel 2011). The ROIs were selected bilaterally from the Nielsen and Hansen's volumes-of-interest defined in the Brainmap database (Nielsen and Hansen 2002). The volumes-of-interest do not provide an anatomical mask for the midbrain including VTA/SN. Therefore, the midbrain mask was defined as a 10 mm sphere centered at an activation peak derived from a previous DDT study (Luo et al. 2009), manually drawn on the standard MNI brain (*x* = 0, *y* = –18, *z* = –13) including bilateral SN and VTA. The a priori defined ROIs are displayed in Figure S2. The REST toolkit was used to correlate the averaged time course within the dACC mask, as the seed time course, to the averaged time course of each ROI using Pearson's correlation analysis. Next, we subjected the Pearson correlation coefficients (*r*) to a Fisher's *Z* transformation to obtain *Z*-scores and improve normality of the data. The averaged time course data of the dACC and Fisher's *Z* correlations of the dACC (rs-FC) with each of the a priori defined ROIs were imported in SPSS (SPSS Inc., Chicago, IL) for further statistical analyses.

Brain morphology was assessed using a Voxel-Based Morphometry toolbox (VBM8; http://dbm.neuro.uni-jena.de/vbm/) with default settings. The VBM8 toolbox is an extension of the unified segmentation model (Ashburner and Friston 2005) in which structural images are bias corrected, segmented into gray matter, white matter, and cerebrospinal fluid, and registered combined within the same model. The proportion of gray matter and white matter within the anatomical mask of the ACC used as the seed region was calculated to correct statistical analyses for tissue composition.

### Statistical analyses

All data were normally distributed. Relationships between dACC Glu/Cr, rs-FC of the dACC with the above mentioned ROIs and delay discounting were explored using bivariate correlation analyses available in SPSS. Because we performed multiple comparisons, we used an adjusted level of *P* < 0.01; we did not adjust the level to reflect all statistical comparisons because this is the first study of this topic and is therefore exploratory. Following correlation analyses, we used the SPSS 17.0 Indirect.sbs script (Preacher and Hayes 2008) to test the indirect effect of X (dACC Glu/Cr) on Y (impulsive decision making) through the mediator M (resting state dACC signal/functional connectivity) using a mediation model described by Baron and Kenny (1986), see Figure S1. To establish mediation, there needs to be a significant relation between X and Y (Fig. S1: path c), between X and M (Fig. S1: path a), and between M and Y (Fig. S1: path b). A significant mediation effect is present when the mediator M reduces or eliminates the effect of X on Y, that is, when the difference (c–c′) is statistically significant. A Sobel test as implemented in Indirect.sbs was used to determine the significance of the mediation effect. In addition, because this method can be biased when used in small samples, we checked with a bootstrapping method with bias-corrected confidence intervals, which is also implemented in Indirect.sbs, the significance of the mediation effect. The bootstrap method is significant if zero is not in the confidence interval. Bootstrap analyses and estimates were based on 10,000 bootstrap samples.

## Results

### Association between delay discounting, glutamate, and resting state functional connectivity

**Figure 2:**
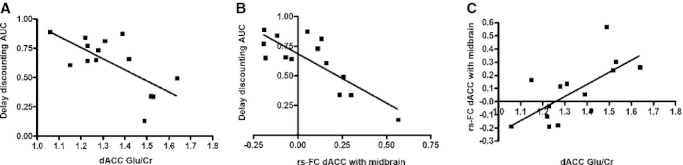
Association between glutamate, rs-FC between dACC and midbrain and delay discounting. Significant negative correlations between (A) left dACC Glu/Cr concentrations and delay discounting, (B) negative correlation between resting state functional connectivity (rs-FC) of the left dACC with the midbrain including VTA and SN and delay discounting, and (C) positive correlation between Glu/Cr concentrations and rs-FC of the left dACC with the midbrain. Smaller delay discounting area under the curve (AUC) values reflect steeper discounting and therefore higher impulsive decision making.

No correlations were found between gray and white matter content of the dACC region corresponding to the ¹H MRS voxel and functional connectivities, Glu ratios or delay discounting values (*P*-values all >0.13), and were therefore not included as a covariate in subsequent analyses. Delay discounting was negatively correlated with Glu/Cr (*r*(14) = –0.68, *P* < 0.01); that is, higher Glu/Cr was associated with steeper discounting of delayed rewards (Fig. 2A). Delay discounting was also negatively correlated with dACC rs-FC with the midbrain including the VTA and SN (*r*(14) = –0.81, *P* < 0.001) (Fig. 2B), but not with the fractional amplitude of low frequency fluctuations (fALFF) within the dACC and rs-FC of the dACC with other ROIs. Glu/Cr was also correlated with rs-FC of the dACC with the midbrain (*r*(14) = 0.68, *P* < 0.01) (Fig. 2C) and with the left (*r*(14) = 0.68, *P* < 0.01) and right PCC (*r*(14) = 0.78, *P* < 0.01). There was no significant correlation between Glu/Cr and fALFF values of the dACC.

### Mediation analysis

**Figure 3:**
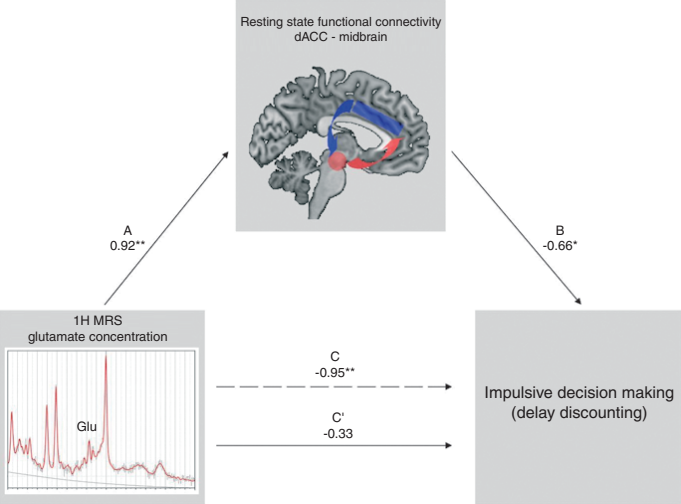
Path analysis. Path analysis showing that the relationship between dACC glutamate concentrations and impulsive decision making (path C) is partially mediated by an increased resting state functional connectivity of the dACC with a midbrain region, including VTA/SN (path C': direct relation between glutamate and delay discounting when corrected for mediator). Sobel test for mediation (path C minus path C'): *Z* = –2.26, *P* = 0.02. The reverse mediation model with dACC glutamate as a mediator of the relationship between resting state functional connectivity of the dACC with the midbrain and impulsive decision making was not significant. *P < 0.01 and **P < 0.001 for path coefficients.

Because delay discounting was associated with both Glu/Cr and rs-FC of the dACC with the midbrain, and Glu/Cr was also correlated with rs-FC of the dACC with the midbrain, mediation analyses were performed to investigate whether dACC Glu/Cr lead to delay discounting through its effect on dACC rs-FC. Mediation analyses showed that the relationship between dACC Glu/Cr and delay discounting was at least partly mediated by an increased functional coupling of the dACC with the midbrain including VTA/SN (Sobel test *Z* = –2.26, *P* = 0.02, see Fig. 3).

### Reverse mediation model

To assess the possibility of reciprocity within the mediation model, that is, that dACC Glu/Cr mediates the relationship between rs-FC of the dACC with the midbrain and DDT scores, we also tested this mediation model. The reverse model did not yield a significant mediation effect of dACC-midbrain connectivity on the association between dACC Glu/Cr and impulsive decision making (Sobel test: *P* = 0.28).

### Controlling for age and IQ

To control for the potential confounding effects of subject age and estimated IQ, partial correlation analyses between dACC Glu/Cr, resting state dACC signal, rs-FC of the dACC, and delay discounting with age and IQ as covariates were carried out. After controlling for age or IQ, dACC Glu/Cr was still significantly correlated with DDT scores, dACC rs-FC with the midbrain, and rs-FC of dACC with the left and right PCC (all *P*-values < 0.04). DDT scores were still significantly correlated with rs-FC of the dACC with the midbrain after controlling for age (*P* = 0.004) and IQ (*P* = 0.002).

## Discussion

Our study is the first to combine evidence from ¹H MRS and rs-fMRI to predict individual differences in impulsive decision making in healthy volunteers. We found evidence that individual differences in impulsive decision making are associated with dACC function under task-free conditions in terms of glutamate concentrations and resting state functional connectivity. In line with previous research (Hoerst et al. 2010), we found that higher impulsivity was associated with higher glutamate concentrations in the dACC. Dorsal ACC glutamate concentrations were also found to be increased in untreated children with ADHD, a disorder characterized by impaired impulse control (Hammerness et al. 2012). In addition, increased functional coupling between the left dACC and a midbrain region including VTA and SN was associated with steeper discounting of delayed rewards, whereas a negative functional coupling was associated with less discounting and therefore lower impulsive decision making. Our findings of an association between resting state functional connectivity between dACC and the midbrain and impulsivity are consistent with a previous study of Tian et al. (2006) who showed increased resting state connectivity between dACC and the midbrain in adolescents with ADHD, a disorder characterized by high levels of impulsivity. In addition, a task-related fMRI study of Diekhof and Gruber (2010) found that preference for immediate rewards was associated with increased functional coupling between the PFC and the midbrain. The stronger interaction between brain regions involved in decision strategy (dACC) and subjective valuation of rewards (midbrain) under rest in high impulsive subjects as observed in the current and above mentioned studies might indicate a potential trait marker for impulsive decision making, especially because low impulsive decision making was associated with no or even a negative coupling between the left dACC and the midbrain. In addition, the current study suggests that this increased functional coupling between the dACC and the midbrain might be driven by glutamate neurotransmission. We established a functional pathway, showing that molecular properties (glutamate concentrations) of the dACC, via functional connectivity of the dACC with the midbrain including VTA and SN, translate into behavioral manifestations of delay discounting. This finding indicates that the relationship between dACC glutamate concentrations and impulsive decision making can, at least partly, be attributed to connectivity of the left dACC with the midbrain.

Preclinical literature has indicated a role for glutamate in impulsivity (for a review see Pattij and Vanderschuren 2008). For instance, selective and nonselective NMDA receptor antagonists have been shown to increase impulsive behavior in animal models (Higgins et al. 2003; Mirjana et al. 2004). Systemic pretreatment with an mGlu2/3 receptor agonist attenuates impulsive behavior seen after serotonin receptor stimulation (Wischhof et al. 2011). In humans, a recent study of Hoerst et al. (2010) examined glutamate levels in the dACC in patients with borderline personality disorder and healthy controls. Irrespective of diagnosis, higher Glu/Cr was associated with higher self-reported (trait) impulsivity (Hoerst et al. 2010). Anterior cingulate Glu/Cr was also found to be increased in untreated children with ADHD, a disorder characterized by impaired impulse control (Hammerness et al. 2012). In keeping with these findings, the current study revealed that glutamate concentrations in the dACC were associated with impulsive decision making.

Interestingly, the current study revealed associations between dACC glutamate concentrations and resting state connectivity of the dACC with other brain regions (the midbrain and the left and right PCC). This is consistent with a previously described correlation between ¹H MRS glutamate concentrations and resting state functional connectivity (Horn et al. 2010), suggesting that the amount of glutamate, which is present in neuronal and glial metabolic and neurotransmitter pools as measured by ¹H MRS, underlies the observed synchronization among these brain regions as assessed with resting state functional connectivity measures. This is not very surprising, as it is thought that the intrinsic energy demands of neuron populations in different brain regions with a common functional purpose have wired together through synaptic plasticity, and thereby form so-called resting state networks (e.g., Lewis et al. 2009). It is well known that glutamate plays a critical role in synaptic plasticity. Unfortunately, ¹H MRS does not allow distinguishing the specific contribution of different components of the glutamatergic system to spontaneous coherence of BOLD signal fluctuations between brain regions. ¹H MRS glutamate measurements reflect primarily intracellular glutamate and does not directly measure synaptic glutamate transmission (Gruetter et al. 1998). Furthermore, quantification and separation of glutamate by use of ¹H MRS is technically difficult because glutamate overlaps in its chemical shift range with glutamine and γ-amino butyric acid (GABA). Although in this study acceptable reliable peaks, as defined by LCModel quality control criteria, were recorded consistently for Glu separate from Glx (total glutamate plus glutamine), undetected (although probably small) contributions from glutamine (Gln) and GABA cannot be ruled out. Future studies are using more advanced spectral editing techniques, such as a spectrally selective refocusing method (Choi et al. 2006) or 2D J-resolved spectroscopy (Jensen et al. 2009), are warranted to separate Glu from Gln. Gln could be of particular interest, as synaptic glutamate taken up by glial cells is converted into glutamine before returning to the presynaptic neuron for conversion back into Glu (Magistretti and Pellerin 1999) and therefore Gln may be a more accurate index of overall glutamatergic neurotransmission than Glu (Rothman et al. 2003).

The ACC is part of a control network, and activity in the ACC correlates with the degree of decision conflict experienced when choosing between an immediate smaller and delayed larger reward (Pochon et al. 2008). Midbrain areas, such as the VTA and SN are involved in the subjective valuation of rewards presented during a DDT (Luo et al. 2009). Recently, it has been shown that the midbrain, through its dopaminergic projection to the striatum, predicts individual differences in impulsivity in humans (Buckholtz et al. 2010). Midbrain dopaminergic neurons project to various brain areas, such as the striatum and PFC, signaling the availability of a reward. The dACC is important in integrating these reward signals in the decision making process, as dACC activation reflects decision conflict and decision strategy (Pochon et al. 2008; Marco-Pallares et al. 2010). Therefore, the current results of associations between functional connectivity between the midbrain and dACC, dACC glutamate concentrations and delay discounting could suggest a projection from the midbrain to the dACC related to signaling reward, thereby increasing activity in the dACC reflected by increased glutamate concentrations, leading to steeper discounting of delayed rewards. However, this should have been reflected by a mediation model with dACC glutamate concentrations as a mediator of the relationship between resting state functional connectivity of the dACC with the midbrain and delay discounting, but this proposed pathway was not significant. Instead, we established a functional pathway from glutamate concentrations in the dACC to delay discounting, through functional coupling between dACC and the midbrain. Rodent studies have indicated the presence of glutamatergic projections from the PFC to the midbrain and it has been suggested that firing of VTA dopamine neurons depends largely on glutamatergic inputs (Kalivas 1993). Evidence of (limited) glutamatergic projections from the ACC to the VTA and SN has also been found in primates (Frankle et al. 2006). The current findings of glutamatergic modulation of resting state functional connectivity of the dACC with the midbrain is suggestive of glutamatergic control of the dACC over midbrain regions, which in turn could modulate midbrain dopamine firing and subsequent dopaminergic projections to the striatum, leading to individual differences in impulsive decision making. However, as ¹H MRS does not directly measure synaptic glutamate transmission, but rather reflects metabolic and neurotransmitter pools of glutamate within a ROI (Gruetter et al. 1998), and resting state functional connectivity does not represent glutamatergic projections from one brain region to another, animal research is needed to further investigate this interpretation of the current findings.

It is important to stress that our results should be viewed in light of some methodological limitations. First, the sample size was modest, which might limit the generalizability of the current results. Second, because of the exploratory nature of our study, we applied a statistical threshold for significance that did not fully account for the issue of multiple comparisons. However, the observed correlation between delay discounting (AUC values) and glutamate concentrations and between delay discounting and functional connectivity of the dACC with the midbrain remained significant when correcting for multiple comparisons. Instead, there was a trend toward a significant correlation between glutamate concentrations and dACC functional connectivity with the midbrain when fully correcting for multiple comparisons. Although the current study was set up as a pilot study and gives a first indication of the interaction between glutamate concentrations and functional connectivity between brain regions under rest in predicting impulsive behavior, future studies are required using larger sample sizes (and thereby increasing statistical power) to replicate the current findings.

In conclusion, the current findings suggest that individual differences in impulsive decision making depend on intrinsic properties of the dACC and not merely on task-related cognitive processes, supporting the idea that altered basic conditions of brain functioning lead to abnormal functional responses. This is important to acknowledge, because spontaneous intrinsic brain processes have been proposed to be a potential promising biomarker of disease (Greicius 2008) and impulsive decision making is a common feature in several psychiatric disorders. Recent findings have indicated that there is a genetic influence on individual differences in delay discounting (Boettiger et al. 2007; Eisenberg et al. 2007; Anokhin et al. 2011). Assuming there is a long road between genes and higher-order cognitive functions, intrinsic properties of brain functioning in the form of resting state glutamate concentrations or functional connectivity, may provide an intermediate step between genes and abnormalities in higher-order cognitive functions, such as excessive delay discounting. In addition, the current study indicates that in addition to previously found involvement of dopaminergic and serotonergic neurotransmission, glutamate signaling plays an important role in human impulsive decision making. Although these findings need to be replicated in larger samples, the current results suggest that glutamate concentrations obtained by ¹H MRS and resting state fMRI are candidate biomarkers for impulsivity and impulsivity related diseases.

## Supporting Information

**Figure S1.** Overview of the a priori defined regions of interest (ROIs) for resting state functional connectivity with the left dACC. ROIs were defined bilateral, but are displayed unilateral. L_PFC, lateral prefrontal cortex; vmPFC, ventromedial prefrontal cortex; PCC, posterior cingulate cortex.

**Figure S2.** The mediation model.
