# Supplementary material for: The association between cingulate cortex glutamate concentration and delay discounting is mediated by resting state functional connectivity
Source: Brain Behav. 2012 Jul 16;2(5):553–62. doi: 10.1002/brb3.74 (PMC3489808; doi:10.1002/brb3.74)
Supplement: Supplementary file 2 [file brb30002-0553-SD2.doc]

The association between cingulate cortex glutamate concentration and delay discounting is mediated by resting state functional connectivity

*Lianne Schmaal*, Anna E. Goudriaan, Johan van der Meer, Wim van den Brink, Dick J. Veltman

**Supporting Information**

**Contents:**

- **Supplementary Figures**

**Supplementary Figures**

*
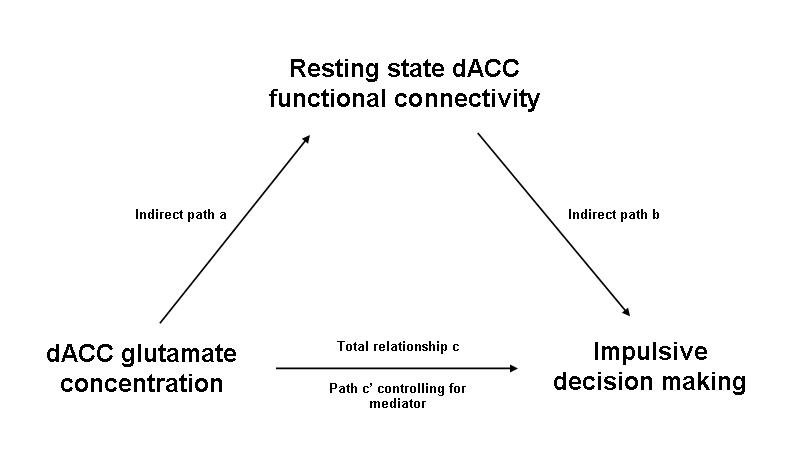
*

**Fig. S2: The mediation model**
